# Unveiling the Role of Schwann Cell Plasticity in the Pathogenesis of Diabetic Peripheral Neuropathy

**DOI:** 10.3390/ijms251910785

**Published:** 2024-10-08

**Authors:** Nurul Husna Abd Razak, Jalilah Idris, Nur Hidayah Hassan, Fazlin Zaini, Noorzaid Muhamad, Muhammad Fauzi Daud

**Affiliations:** 1 Institute of Medical Science Technology, Universiti Kuala Lumpur (UniKL), A1-1, Jalan TKS 1, Taman Kajang Sentral, Kajang 43000, Selangor, Malaysia; husna.razak02@s.unikl.edu.my (N.H.A.R.); jalilahidris@unikl.edu.my (J.I.); nurhidayah@unikl.edu.my (N.H.H.); 2 Royal College of Medicine Perak, Universiti Kuala Lumpur (UniKL), No. 3, Jalan Greentown, Ipoh 30450, Perak, Malaysia; fazlin@unikl.edu.my (F.Z.); noorzaid@unikl.edu.my (N.M.)

**Keywords:** diabetic peripheral neuropathy (DPN), Schwan cell plasticity, neurodegenerative disease

## Abstract

Diabetic peripheral neuropathy (DPN) is a prevalent complication of diabetes that affects a significant proportion of diabetic patients worldwide. Although the pathogenesis of DPN involves axonal atrophy and demyelination, the exact mechanisms remain elusive. Current research has predominantly focused on neuronal damage, overlooking the potential contributions of Schwann cells, which are the predominant glial cells in the peripheral nervous system. Schwann cells play a critical role in neurodevelopment, neurophysiology, and nerve regeneration. This review highlights the emerging understanding of the involvement of Schwann cells in DPN pathogenesis. This review explores the potential role of Schwann cell plasticity as an underlying cellular and molecular mechanism in the development of DPN. Understanding the interplay between Schwann cell plasticity and diabetes could reveal novel strategies for the treatment and management of DPN.

## 1. Introduction

Diabetic peripheral neuropathy (DPN) is a disorder that affects peripheral nerves as a result of diabetes. It is the most common complication among diabetic patients, affecting approximately 30% of them [1,2]. The symptoms of DPN include numbness, tingling, and weakness [3,4]. These conditions typically manifest in the feet and hands in a bilateral and symmetric pattern, often referred to as the “stocking and glove” distribution of neuropathy [5]. Additionally, DPN patients frequently experience neuropathic pain, which can severely impact their functionality, mood, sleep, and overall mental health [6,7]. In advanced stages of diabetic neuropathy, patients may exhibit motor weakness and multiple-organ dysfunction due to damage to motor and autonomic nerves [4,8].

**Figure 1:**
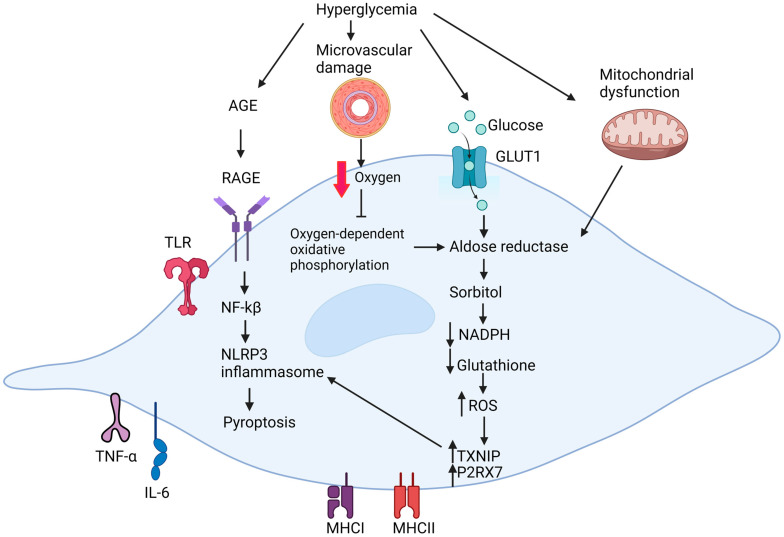
Summary of the pathogenic mechanism in Schwann cells under diabetic conditions. Created with BioRender.com.

The pathogenesis of diabetic peripheral neuropathy is associated with axonal atrophy, Schwann cell demyelination, reduced regenerative capacity, and peripheral nerve fiber loss [9]. However, the precise cellular and molecular mechanisms underlying this pathogenesis remain unclear. The major hypotheses describing the pathophysiological mechanisms of DPN include metabolic dysfunction, microvascular damage, and inflammatory dysregulation (Figure 1).

Firstly, hyperglycemia induces metabolic dysfunction by impairing the mitochondrial function and reducing the glycolytic flux, leading to hyperactivity of the polyol pathway. This results in increased reactive oxygen species (ROS) and advanced glycation end-product (AGE) formation, contributing to oxidative stress. Secondly, diabetes-induced microvascular damage disrupts the endoneurial blood flow, creating hypoxic conditions in the neural microenvironment and exacerbating oxidative damage through metabolic dysfunction. Thirdly, inflammatory dysregulation, triggered by hyperglycemia and dyslipidemia, leads to the accumulation of AGEs and elevated levels of modified low-density lipoproteins (LDLs). This, in turn, causes endoplasmic reticulum (ER) stress, mitochondrial damage, and increased ROS generation in nerve tissue.

Recent research on diabetic neuropathy has largely adopted a neuron-centric approach to understand its pathogenic development [10,11]. However, much less attention has been given to the response of Schwann cells under diabetic conditions. Schwann cell impairment is often considered a secondary effect of axonopathy. Nevertheless, several findings suggest that Schwann cells may play a crucial, if not primary, role in diabetic neuropathy. This idea is thoroughly explored in the review by Wu et al., 2024 [12]. The unique plasticity of Schwann cells has been well characterized, particularly in the context of peripheral nerve injury and regeneration [13]. This plasticity allows Schwann cells to undergo phenotypic reprogramming, shifting from a mature/myelinating state to a repair state in response to pathophysiological conditions [14].

It is therefore unsurprising that the peripheral nervous system has a relatively superior regenerative capacity compared to the central nervous system [15,16]. While this remarkable cellular reprogramming facilitates the creation of regenerative environments following injury, it also renders Schwann cells susceptible to demyelinating diseases due to phenotypic instability in response to slight disturbances in cellular or tissue homeostasis [17]. The diabetic-induced alteration of Schwann cell plasticity has yet to be comprehensively elucidated. This review will focus on the role of Schwann cells in the pathogenesis of diabetic neuropathy, with particular emphasis on the mechanisms by which diabetic conditions may disrupt Schwann cell plasticity. For a comprehensive discussion on the overall mechanisms of peripheral nerve injury and repair, readers may refer to a review by Menorca et al., 2013 [18].

## 2. Schwann Cell Roles in Peripheral Nerves

**Figure 2:**
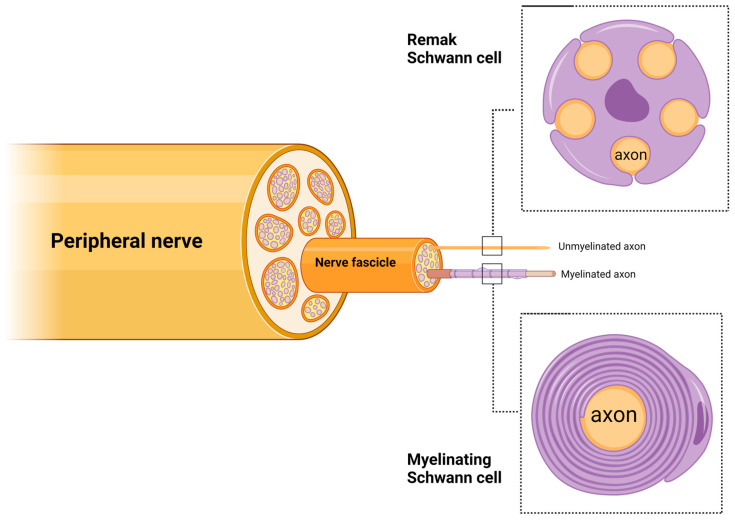
The organization of Remak Schwann cells and myelinating Schwann cells in peripheral nerves. Created with BioRender.com.

Schwann cells (SCs) are the most abundant glial cells in the peripheral nervous system. Schwann cells are derived from neural crest cells and differentiate into adult Schwann cells via two intermediate stages: Schwann cell precursors in the embryonic stage and immature Schwann cells in the late embryonic and perinatal nerves [19,20]. Then, immature Schwann cells undergo radial sorting by extending their cytoplasmic projections into bundles of axons to differentiate into myelinating or Remak Schwann cells (Figure 2) [21]. Schwann cells are intimately associated with axons by enwrapping them to form either the myelin sheath or the Remak bundle. Myelinating Schwann cells form the myelin sheath via their plasma membrane, which wraps the axon in segments, separated by the nodes of Ranvier. This is responsible for increasing the conduction speed of the action potential. The myelin sheath has a low capacitance and subsequently reduces the membrane electrical resistance across internode intervals to allow for rapid, saltatory nerve impulses from node to node [22]. This process is unique in myelinated axons, as myelin serves as an electrical insulator. The nodes of Ranvier are arranged as gaps in the myelin sheath. The axonal membrane at the nodes of Ranvier is exposed to the extracellular space and is enriched in sodium and potassium ion channels [23]. Through this unique process, many myelinated axons with high conduction speed can be placed in a limited space to permit the development of a complex nervous system, whereas unmyelinated axons would require a larger area. Saltatory conduction eliminates the need to regenerate the action potential at every point of the axonal membrane. This indirectly reduces the metabolic requirements for neuronal activity [22]. Thus, myelin increases the conduction speed and reduces space and energy for nerve impulse conduction [24].

Remak Schwann cells are non-myelinating Schwann cells that wrap multiple small-diameter axons in Remak bundles [25]. Remak Schwann cells are essential for the normal peripheral nervous system (PNS) development and function and assist in regeneration after peripheral nerve injury [26]. However, Remak Schwann cells have not been extensively studied because of the need for electron microscopy to observe these cells and the lack of specific markers to distinguish between Remak Schwann cells and immature Schwann cells [26].

Schwann cells support axons during development, homeostatic maintenance, and regeneration through various means (reviewed by [27]). Schwann cells provide trophic support through the release of a plethora of neurotrophic molecules such as nerve growth factor, brain-derived growth factor, ciliary neurotrophic factor, vascular endothelial growth factor, hepatocyte growth factor, neurotrophin-3, pleiotrophin, and insulin-like growth factor [27]. Schwann cells’ trophic molecules also provide neuroprotection by limiting axonal degeneration and promoting neuronal survival following injury [28]. Schwann cells also exert their neuroprotective effect on axons through exosome transfer, which helps to inhibit neuronal apoptosis by attenuating inflammation [29] and blocking the caspase-3 cell death pathway [30]. Schwann cells provide material supplies to axons through the transfer of polyribosomes to support axonal protein synthesis [31]. Axons also receive iron and lactate from Schwann cells for axonal metabolic functions [32,33].

The maintenance of axonal integrity is closely linked to Schwann cell metabolic support. Thus, even though Remak Schwann cells do not myelinate, their mitochondria are crucial for maintaining axonal integrity. The dysfunction of Schwann cell mitochondria results in several peripheral neuropathies. The deletion of the mitochondrial transcription factor A (Tfam) in embryonic Schwann cells results in the degeneration of unmyelinated axons and the consequent loss of myelinated axons, even though Schwann cells survive [34]. This study observed that axonal degeneration was not secondary to neuronal death, as neuronal cell bodies did not exhibit elevated levels of apoptotic markers. This finding highlights the significance of Schwann cell metabolic activities in axoglial interactions for maintaining axonal integrity [34]. In addition, the serine/threonine kinase LKB1 modulates Schwann cell-mediated maintenance of axons via its functions in Schwann cell polarity and mitochondrial metabolism [35,36,37]. Delayed myelin initiation, hypomyelinated nerves, and impaired ensheathment of Remak Schwann cells were observed, and behavioral symptoms of neuropathy were observed in animal models when LKB1 was deleted in embryonic Schwann cells [36,37]. Through these studies, the disruption of Schwann cell metabolism caused the loss of unmyelinated axons, indicating that even unmyelinated axons may depend on glial support.

## 3. Schwann Cell Plasticity during Tissue Regeneration

**Figure 3:**
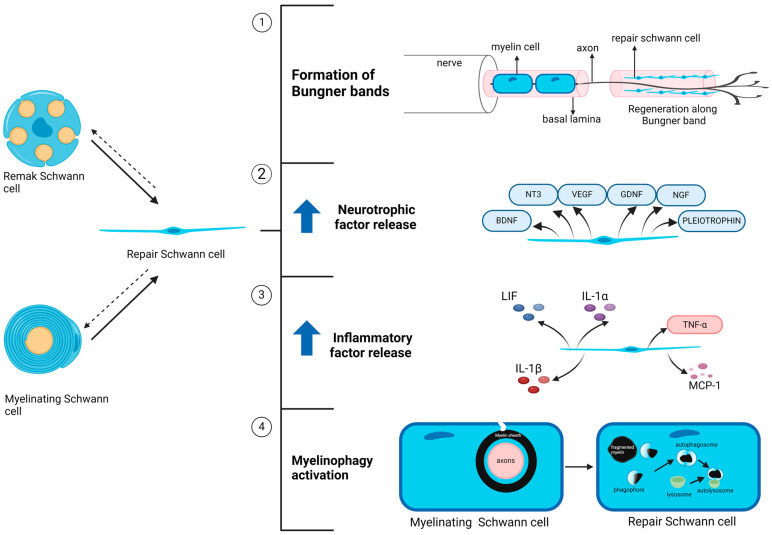
The role of Schwann cell plasticity in nerve regeneration. Following injury, both myelinating and Remak Schwann cells can reversibly transform into Repair Schwann cells due to the plasticity of Schwann cell phenotype. Repair Schwann cells facilitate peripheral nerve regeneration by forming the band of Bungner (1), elevating release of neurotrophic (2) and inflammatory factors (3) and promoting myelinophagy (4). Created with BioRender.com.

The peripheral nervous system (PNS) has a better regenerative capacity than the central nervous system (CNS). Numerous studies have demonstrated that Schwann cells play a vital role in axonal regeneration [38]. This is attributed to the plasticity of Schwann cell phenotypes, as these cells can transform from a mature, maintenance phenotype into a proliferative, pro-regenerative phenotype (Figure 3) [13]. After injury, both myelinating and non-myelinating Schwann cells, which are normally mitotically inactive, re-enter the cell cycle and undergo mitosis to proliferate rapidly after converting into repair Schwann cells [39,40]. They become elongated and align in layers upon one another inside each of the endoneurial tubes, which were formerly occupied by axons and mature Schwann cells, and subsequently form a structure known as bands of Bungner. This structure aids in the formation of guiding tracks for regenerating axons, directing the latter to the nerve target areas through structural and biochemical support [41]. Interestingly, almost 50% of Remak Schwann cells reprogram into repair Schwann cells and build Bungner bands to assist the regeneration of axons. They can also differentiate into myelinating Schwann cells after regeneration [42].

As part of the Schwann cell injury response, neurotrophic factors such as glial cell line-derived neurotrophic factor (GDNF), artemin, brain-derived neurotrophic factor (BDNF), neurotrophin-3 (NT3), nerve growth factor (NGF), vascular endothelial growth factor (VEGF), and pleiotrophin are upregulated in repair Schwann cells to promote the survival of injured axons and axonal elongation [40,43,44,45,46,47]. In addition, the expression of cytokines, including tumor necrosis factor (TNF)-α, leukemia inhibitory factor (LIF), interleukin (IL)-1α, IL-1β, and monocyte chemoattractant protein-1 (MCP-1), is increased [48,49]. MCP-1 is a cytokine that recruits macrophages, which are critical for Wallerian degeneration [50,51]. The opening of the blood–nerve barrier, triggered by the release of serotonin, histamine, and other substances from resident mast cells, facilitates macrophage invasion. Macrophages are essential for helping Schwann cells clear myelin debris [52]. They facilitate the clearing of myelin debris because they can phagocytose myelin debris in the extracellular spaces or directly in Schwann cells’ cytoplasm [53].

Myelin clearance is vital in the repair process because myelin debris inhibit axonal growth, directly contributing to the failure of axonal regeneration [54]. Schwann cells begin to break down their own myelin and distal axon segments in the early stages of Wallerian degeneration. After 24–48 h of nerve injury, myelin destruction begins, where the myelin sheath is fragmented into myelin ovoids, stored in the cytoplasm of Schwann cells called digestion chambers [55,56]. Fragments of the axoplasm can also be observed in the digestion chambers as Wallerian degeneration progresses. In the following days, the myelin ovoids are further disintegrated and cleared with the help of macrophages [53]. Schwann cells can break down their myelin owing to the activation of the cell-intrinsic myelin breakdown process through autophagy, termed myelinophagy [57,58]. After clearing myelin debris and axon debris, macrophages are removed from the nerve by local apoptosis and re-enter the circulation to move to the regional lymph node and spleen [59]. For a comprehensive discussion on the roles of Schwann cells in nerve injury repair, readers are encouraged to consult the review by Nocera and Jacob, 2020 [13]

## 4. Pathological Changes in Schwann Cell under Diabetic Conditions

This section discusses the molecular mechanisms of diabetic neuropathy, with a specific focus on the role of Schwann cells. The interactions between neurons and Schwann cells in diabetic neuropathy have been comprehensively reviewed by Gonçalves et al., 2017 [60]

### 4.1. Metabolic Dysfunction in Diabetic Schwann Cells

The exact pathogenic progression of diabetic neuropathy remains under an open investigation. Thus far, it is clear that oxidative stress resulting from excessive reactive oxygen species (ROS) generated through the hyperactivation of the polyol pathway plays a central role in disease development (Figure 1) [61]. The polyol pathway involves two steps. Aldose reductase (AR) reduces glucose to sorbitol and consumes nicotinamide adenosine dinucleotide phosphate (NADPH). Sorbitol dehydrogenase (SDH) then catalyzes the oxidation of sorbitol to fructose, with nicotinamide adenine dinucleotide (NAD^+^) as a cofactor [62,63]. Schwann cells are particularly susceptible to oxidative damage as they are the primary ROS production site within the endoneurium because aldose reductase is highly expressed in Schwann cells [64].

The hyperactivation of the polyol pathway is linked to the overproduction of free radicals caused by the imbalanced consumption of NAD + and NADPH, which are essential metabolic cofactors. AR activity consumes NADPH to metabolize glucose into sorbitol via the polyol pathway. Since NADPH is jointly used by glutathione reductase (GR) to reduce glutathione disulfide (GSSG) into glutathione (GSH), an endogenous antioxidant [65], elevated AR activity inhibits GR activity, reducing intracellular GSH and, as a result, enhancing oxidative stress [66]. Moreover, the second reaction in the polyol pathway further exacerbates oxidative stress by promoting the generation of superoxide anions. During the oxidation of sorbitol to fructose, NAD+ is concomitantly reduced to NADH. The elevation in NADH within cells boosts the activity of NADH oxidase and the production of superoxide anions [67].

In addition, the depletion of cytosolic antioxidants, such as taurine, may contribute to oxidative and nitrosative stress. The link between hyperglycemia and taurine depletion was previously demonstrated in the nerve tissue of a diabetic rat model [68]. Taurine helps to counter hyperglycemia-induced nitrosative stress by downregulating the expression of inducible nitric oxide synthase (iNOS) and neuronal NOS (nNOS) [69]. However, polyol-driven sorbitol accumulation creates osmotic pressure that expels taurine from Schwann cells [68]. Moreover, taurine transport is adversely affected by diabetes. Under hyperglycemic stress, human Schwann cells downregulate taurine transporters and reduce taurine uptake by at least 40% and 30%, respectively [70], potentially due to nitrosative stress [69]. Taurine transport functions were improved after treatment with sorbinil, an AR inhibitor, as demonstrated in a human Schwann cell culture study [70], thus indicating the role of the polyol pathway in hyperglycemia-induced taurine depletion.

The accumulation of advanced glycation end products (AGEs) is another causal factor in the development of oxidative stress in diabetes [71]. AGEs can accumulate in cells and the extracellular matrix through the glycation of nucleotides, lipids, and proteins, which alters their structure and function. Excess glucose and fructose accumulation increase the production of α-dicarbonyl compounds (α-DCs) such as methylglyoxal (MGO), glyoxal (GO), and 3-deoxyglucosone (3DG), and these compounds react randomly with DNA, lipids, and proteins to generate AGEs. The activation of the receptor for advanced glycation end products (RAGE) leads to neuronal injury by promoting oxidative stress through the upregulation of phosphatidylinositol-3 kinase activity [72]. While it is not clear whether Schwann cells undergo oxidative damage following RAGE activation, Schwann cells derived from patients with diabetic polyneuropathy exhibited a significant increase in RAGE signaling activity [73]. Additionally, a previous study showed that the antioxidant α-lipoic acid could reverse the adverse effects of AGEs on Schwann cells, such as impaired growth and apoptosis [74]. This finding suggests a possible link between AGEs and ROS formation in Schwann cells.

Moreover, mitochondrial dysfunction induced by hyperglycemia may contribute to the overactivation of the polyol pathway. Mitochondrial damage has been observed in the sciatic nerve of a diabetic rodent model, characterized by the upregulation of multiple subunits of complexes I, III, IV, V and mitochondrial Rho GTPase 1 [75]. Similarly, SILAC analysis of primary Schwann cell cultures has shown that hyperglycemia alters the mitochondrial proteome and reduces mitochondrial efficiency [76]. Additionally, diabetes may impair glycolytic functions, as reduced glycolysis–TCA cycle flux has been reported in IMS32 Schwann cells under hyperglycemic conditions [77]. Mitochondrial dysfunction and impaired glycolytic flux may prompt Schwann cells to shift their energy production functions by enhancing collateral glycolysis pathways, such as the polyol pathway [78], thereby driving the pathogenesis of diabetic neuropathy.

### 4.2. Effect of Microvascular Damage on Schwann Cells

It is thought that microvascular damage precedes oxidative damage in Schwann cells [79,80,81]. Endoneurial capillary damage, caused by exposure to increased levels of advanced glycation end products (AGEs), manifests through basement membrane thickening, loss of pericyte coverage, and endothelial hyperplasia (Figure 1) [81]. These damages may lead to capillary dysfunction, disturbing the endoneurial blood flow patterns and, in turn, limiting the extraction of oxygen by the endoneurial tissue because of the short capillary transit time [82]. Schwann cells then switch their mode of energy production from oxygen-dependent oxidative phosphorylation to the polyol pathway in response to hypoxic conditions. As a result, they generate more ROS and further exacerbate the oxidative damage.

Notwithstanding the above, it is noteworthy that there are previous studies that contradict the microvascular damage hypothesis. For instance, animal studies demonstrated elevated endoneurial blood flow immediately after diabetic induction [83], while patients with mild diabetes showed no progressive sural nerve blood flow deterioration over one year [84].

### 4.3. Inflammatory Dyregulation in Diabetic Schwann Cells

It is now a consensus that inflammation plays a vital role in the pathogenesis of diabetic neuropathy. Inflammation may occur relatively early in the pathogenic development and persist throughout the process, which makes it an attractive target for therapeutic strategies. Various preclinical and clinical studies have demonstrated a significant link between the inflammatory response and DN pathogenesis. For instance, the nerve tissue of diabetic patients exhibits tissue repair responses by increasing the expression of genes associated with axonogenesis, inflammatory responses, and injury responses [85]. Several clinical studies have also reported a high degree of association between diabetic peripheral neuropathy and elevated plasma levels of pro-inflammatory cytokines, such as C-reactive protein (CRP), interleukin 6 (IL-6) [86], tumor necrosis factor-alpha (TNF-α) [87,88], and transforming growth factor-beta 1 (TGF-β1) [89]. The upregulation of proinflammatory cues corresponds to the activation of various immune cells. A previous study by G. Conti et al. observed macrophage infiltration in the sciatic nerve of diabetic rats using a single-fiber immunostaining method with the ED1 monoclonal antibody [90]. Macrophage infiltration may contribute to neuropathy, as macrophages, through cytokine secretion, are directly involved in peripheral nervous system demyelination and remyelination inhibition [91]. Increased neutrophil infiltration has also been observed in the spinal cord parenchyma of diabetic rodents due to increased L-selectin levels and spinal cord vasculature. L-selectin is a vital adhesion molecule involved in neutrophil transmigration [91].

The infiltration of immune cells, such as macrophages, into diabetic nerve tissue may be regulated by Schwann cells through the release of chemokines and cytokines, including monocyte chemotactic protein-1 (MCP-1), macrophage inflammatory protein-1 alpha (MIP-1α), TNFα, and IL-1α, as demonstrated in nerve injury models [92] Once macrophages enter the nerve tissue, they collaborate with Schwann cells to release these cytokines, thereby amplifying the recruitment of additional macrophages into the area [93]. In nerve injury responses, the release of cytokines by Schwann cells is typically stimulated by products of degenerated neural tissue [94]. However, in the context of diabetes, it is not entirely clear how the initiation of cytokine expression begins. It is possible that classical diabetic stress signals, such as advanced glycation end products (AGEs) and reactive oxygen species (ROS), may trigger this process.

The neuroinflammatory functions of Schwann cells are well recognized, especially in the context of peripheral nerve injury and neurodegenerative diseases such as amyotrophic lateral sclerosis (ALS). Their roles are integral to the peripheral nerve tissue’s innate and adaptive immune systems, making Schwann cells highly immune-competent [95]. Schwann cells express various pattern recognition receptors (PRRs), which are commonly found in professional innate immune cells, such as macrophages and natural killer cells, to detect pathogens. PRRS expressed in Schwann cells includes Toll-like receptors (TLRs) [96], the nucleotide-binding and oligomerization domain (NOD)-like receptor (NLR) family [97], the receptor for advanced glycation end products (RAGE) [98], the mannose receptor (MR) [99], the C-type lectin receptor (CTR) [100], and low-density lipoprotein receptor-related protein-1 (LRP-1) [101]. In addition, Schwann cells elicit adaptive immune functions by activating T cells in peripheral nerves. Schwann cells promote T cell activation via the expression of major histocompatibility complex (MHC) classes I and II [102,103,104]. In addition, Schwann cells can also exhibit secondary signals to regulate T cell activity by expressing co-stimulatory molecules such as CD80, ICAM1, and CD74 [105,106].

Diabetic stress triggers inflammatory pathogenesis in Schwann cells on multiple fronts, ranging from the accumulation of AGEs and elevated levels of modified low-density lipoproteins (LDLs) and danger-associated molecular pattern (DAMP) molecules to mitochondrial dysfunction, ER stress, and ROS generation (Figure 1). Stress signaling probably manifests its effects simultaneously during the pathogenic development, thus amplifying the pathogenic downstream impact. Intracellularly, these signals converge and activate NLRP3 inflammasomes, which are cytoplasmic multimeric protein complexes that are central to the inflammatory cascades [107]. NLRP3 inflammasome activation is a two-stage process [108]. The first stage is the priming step, where nuclear factor-κβ (NF-κβ) is activated, which is followed by the activation step that involves the gathering of the NLRP3 inflammasome, triggered by stimuli from multiple cellular and molecular events, including K^+^ efflux, Ca^2+^ signaling, and ROS generation. Through the assembly of the NLRP3 inflammasome, pro-caspase-1 undergoes changes to cleaved-caspase-1 and subsequently promotes the cleavage of gasdermin D (GSDMD) to the N-terminus of GSDMD (GSDMD-NT) and increases the cleavage of pro-IL-1β and pro-IL-18 precursors to mature forms. GSDMD-NT is responsible for the formation of pores in the plasma membrane of cells, resulting in programmed inflammatory cell death or pyroptosis.

In diabetic Schwann cells, stress stimuli such as AGEs and HMGB1 activate the RAGE and TLR-4 signaling pathways, promoting the nuclear translocation of NF-κB and the subsequent priming of the NLRP3 inflammasome. In addition, endoplasmic reticulum (ER) stress, mitochondrial damage, and increased ROS generation [109,110], driven by the hyperactivity of the polyol pathway, elevate thioredoxin-interacting protein (TXNIP) activity, and TXNIP binds to NLRP3 protein and activates the inflammasome pathway [111,112]. In addition, the excessive activation of P2RX7, another consequence of ROS overproduction, also acts as a parallel signaling for NLRP3 inflammasome activation [107]. P2RX7 is a purinergic P2 receptor that can be activated by high concentrations of extracellular adenosine 5′-triphosphate (ATP) in various pathogenic conditions, such as nerve injury [113], diabetes, and depression [114]. Increased inflammasome activity leads to Schwann cell death through pyroptosis [107].

## 5. Schwann Cell Dysfunctions Compromise the Functional Integrity of Peripheral Nerves in Diabetic Neuropathy

The normal neurological function depends on the ability of the nerve tissue to transmit action potentials along neural pathways and maintain the nerve conduction velocity—the speed at which these impulses travel. This capability is largely attributed to Schwann cells and their myelin sheath structures. The disintegration of the myelin sheath can lead to a decline in nerve conduction velocity due to the failure to maintain electrical insulation and establish saltatory conduction. A slowing of nerve conduction velocity is commonly observed in diabetic patients [115,116], and this abnormal nerve conduction is associated with Schwann cell demyelination [112,117]. Two potential mechanisms explain how diabetes dysregulates Schwann cell function and causes demyelination: (1) through the necroptotic pathway; (2) through the apoptotic pathway.

The necroptotic pathway contributes to demyelination via mixed-lineage kinase domain-like protein (MLKL), a key regulatory protein in necroptosis. In its inactive form, MLKL loosely associates with the membrane of Schwann cells. Upon activation through phosphorylation at the serine 441 site, MLKL becomes an integral membrane protein and inserts into the myelin sheath, causing myelin decompaction [118]. While MLKL activation in Schwann cells can be triggered by nerve injury, recent evidence suggests that diabetic stress also promotes MLKL activity. For instance, a study by Guo et al. demonstrated that diabetic mice lost almost 50% of intact myelin sheaths, with nerve conduction reduced by at least 30%, due to the direct effects of upregulated MLKL activity under diabetic conditions [117]. The same study observed elevated MLKL activation in the myelin sheaths of patients with diabetic neuropathy [117].

Conversely, the apoptotic pathway impairs Schwann cell function through the activity of TXNIP. Under hyperglycemic conditions, the TXNIP levels are elevated in Schwann cells, promoting apoptosis and reducing autophagy. This leads to the disintegration of the myelin sheath and impaired nerve conduction [119,120]. Previous studies showed that diabetic mice exhibited significant nerve conduction deficits, with a reduction in nerve conduction velocity by nearly 30%, due to elevated TXNIP expression in Schwann cells [112].

## 6. Dysfunctional Schwann Cell Plasticity in Diabetic Neuropathy: Diabetes-Induced Schwann Cell Reprogramming into Repair Phenotypes

Schwann cell impairment is a key feature of diabetic neuropathy; however, the role of Schwann cell plasticity in the pathogenic development remains unexplored. The remarkable plasticity of Schwann cells helps to create a favorable microenvironment for axonal regeneration and tissue repair. Following injury, Schwann cells reprogram into a repair phenotype that exhibits pro-regenerative properties. We argue that dysfunction of Schwann cell plasticity mediates diabetes-induced nerve injury. Here, we present current findings that support our position.

### 6.1. Phenotypic Alteration in Diabetic Schwann Cells Resembles the Nerve Injury Response

During the early stages of the tissue injury response, nerve tissues experience significant demyelination as Schwann cells lose contact with axons. Interestingly, similar demyelination is observed in diabetic peripheral nerves, even when the axonal membranes remain intact. Studies on diabetic models, including teased fiber analyses of sural nerves from both humans and small animals, have shown that the myelin sheath undergoes morphological deterioration—ranging from thinning to full segmental demyelination—even in the presence of normal axons [121,122,123,124,125]. The degradation of the myelin sheath [123] and the heightened inflammatory response [90,126,127] in diabetic nerves strongly resemble Wallerian degeneration. This suggests that diabetic stress stimuli may trigger the activation of the Schwann cell repair phenotype, much like injury stimuli regulate Schwann cell plasticity.

Additionally, protein and gene expression analyses of sciatic nerves in diabetic rats confirmed a significant reduction in the levels of myelin proteins, such as myelin-associated glycoprotein (MAG), myelin basic protein (MBP), and protein zero (P0) [128,129]. Moreover, studies have shown that diabetes leads to a concomitant increase in P75 expression, a marker of immature Schwann cells. Similar findings have been replicated in an in vitro model using the IMS32 Schwann cell line, where diabetic conditions upregulated P75NTR gene expression while downregulating the expression of the MAG, MBP, and P0 genes [128]. The shift from a mature, myelinated phenotype to an immature state indicates that diabetes can induce cellular reprogramming in Schwann cells, causing them to lose their differentiated characteristics. Given that repair Schwann cells share a similar protein expression profile with immature Schwann cells, it is highly likely that diabetes promotes the reprogramming of Schwann cells into a repair phenotype [13]. Furthermore, since the cellular and tissue responses to diabetes closely mirror nerve injury mechanisms, repair Schwann cells are more likely to be present in diabetic nerves than immature Schwann cells, which are primarily found during development.

### 6.2. Diabetes Elevates the Activity of Repair Regulatory Pathways in Schwann Cells

Several regulatory pathways associated with repair Schwann cells are upregulated in diabetic peripheral neuropathy. First, in patients with type-2 diabetes, elevated plasma glucose levels activate c-Jun N-terminal Kinase (JNK) [130]. JNK, a serine/threonine kinase of the mitogen-activated protein kinase (MAPK) family, plays a central role in regulating cellular stress responses. One key downstream target of JNK is the c-Jun transcription factor [130]. Although repair Schwann cells share molecular characteristics with immature cells, the activation of c-Jun is specific to repair Schwann cells [131]. Myelination is controlled by the antagonistic relationship between c-Jun, a negative regulator, and Krox-20, a promoter of myelination [132]. Krox-20 promotes myelination partly by suppressing the JNK pathway, thereby reducing c-Jun activity and preventing the expression of repair phenotypes in Schwann cells [133,134]. A recent study on diabetic mice demonstrated a significant increase in the nuclear distribution of phospho-c-Jun in myelinating Schwann cells [135], confirming that fully differentiated Schwann cells can reprogram into repair phenotypes under diabetic conditions.

The activation of ERK signaling provides additional evidence that Schwann cells undergo reprogramming into repair phenotypes under diabetic conditions. ERK phosphorylation levels were shown to increase significantly when Schwann cells were exposed to hyperglycemia [136]. Furthermore, elevated ERK levels are associated with reduced myelin protein expression in Schwann cells cultured under hyperglycemic conditions [137]. The ERK signaling pathway plays a pivotal role in controlling Schwann cell plasticity during peripheral nerve regeneration. Previous studies showed that this pathway promotes the expression of repair phenotypes in Schwann cells even in the absence of axonal damage in diabetic in vivo models [138].

The neuregulin1 (NRG1)/ErbB signaling system is also believed to regulate Schwann cell plasticity due to its involvement in a wide range of Schwann cell functions [138]. The NRG1/ErbB pathway controls essential processes such as Schwann cell survival, proliferation, migration, differentiation, myelination, and demyelination [138,139]. The diverse roles of this pathway in Schwann cell biology are partly due to the differential expression of NRG1 isoforms. For instance, NRG1 type III promotes myelination [140], whereas increased expression of NRG1 types I and II leads to Schwann cell dedifferentiation and demyelination [141,142]. A study in diabetic mice revealed that hyperglycemia can alter the expression of NRG1 isoforms, with a decrease in NRG1 type III and an increase in NRG1 type I in the sural nerves of diabetic mice [139]. These changes in NRG1 signaling, which promote demyelination, suggest that repair Schwann cell reprogramming may be activated under diabetic conditions.

## 7. Failure of Repair Schwann Cells to Rescue Tissue Damage in Diabetic Neuropathy

If diabetes triggers the reprogramming of Schwann cells into a repair state, why does this increased repair activity fail to establish a pro-regenerative environment that can reverse nerve damage in diabetic peripheral neuropathy? One possible explanation is impaired myelinophagy in these repair Schwann cells. Myelinophagy, a critical function of Schwann cells during Wallerian degeneration, clears myelin debris and promotes axon regeneration. In injured nerves, myelinophagy is positively regulated in repair Schwann cells via the JNK/c-Jun pathway [57]. However, under hyperglycemic conditions, Schwann cell autophagy can be inhibited. Studies in diabetic mice have shown a significant decline in autophagy, evidenced by reduced levels of autophagy markers such as LC3-II/LC3-I and P62, along with defects in myelinated nerve fibers [120]. This finding is further supported by in vitro studies using RSC96 cells and primary rat Schwann cells, which demonstrate that hyperglycemia impairs Schwann cell autophagy through JAK-STAT3 signaling [120]. Schwann cell autophagy is essential for breaking down myelin components and the degradation of the myelin sheath [57]. Consequently, under diabetic conditions, impaired myelinophagy in repair Schwann cells may result in the accumulation of myelin debris, which hinders nerve tissue repair by inhibiting axonal growth [143].

## 8. Conclusions

Although diabetic peripheral neuropathy (DPN) has traditionally been viewed primarily as a neuronal disorder, recent evidence suggests that Schwann cell impairment plays a critical role in its pathogenesis. In this review, we proposed the hypothesis that the dysregulation of Schwann cell plasticity also contributes to the development of diabetic neuropathy. The plasticity of Schwann cells, which allows these cells to transition between myelinating and repair phenotypes, is disrupted under diabetic conditions. Diabetic stress leads to altered gene expression and cell signaling pathways in Schwann cells, resulting in demyelination and ultimately contributing to nerve damage and sensory dysfunction in DPN patients. The changes observed in diabetic Schwann cells are comparable to those seen in repair Schwann cells following nerve injury.

The current evidence presented in this review supports the hypothesis that Schwann cell plasticity is implicated in the pathogenesis of diabetic neuropathy. However, it is important to note that comprehensive and systematic studies exploring the alteration of Schwann cell plasticity in diabetic neuropathy remain limited. Therefore, the question of whether Schwann cell plasticity is directly involved in the pathogenesis of diabetic neuropathy requires further investigation. If so, other critical questions arise, as follows: How exactly does Schwann cell plasticity regulate the pathogenic mechanisms of DPN? Is the reprogramming of repair Schwann cells a consequence of metabolic and inflammatory dysfunction in diabetic Schwann cells, as well as of microvascular damage? Or do repair Schwann cells drive the pathogenic development of the disease? These questions necessitate further exploration of the interplay between the molecular mechanisms of DPN and the regulatory pathways of Schwann cell plasticity.

Overall, further research on Schwann cell plasticity and its role in DPN is warranted. By elucidating the mechanisms underlying the dysfunction of Schwann cell plasticity in diabetes, we may uncover novel therapeutic strategies to prevent or mitigate the progression of DPN and improve the quality of life of millions of individuals affected by this condition.

## Data Availability

Not applicable.
